# Prevalence and Genomic Characterization of ESBL-Producing *Escherichia coli* in Livestock and Farmers in Catalonia, Spain

**DOI:** 10.3390/antibiotics15070676

**Published:** 2026-07-09

**Authors:** Marina Serras-Pujol, Noemí Párraga-Niño, Marian Navarro, Anna Vilamala, Emma Puigoriol, Elisenda Arqué, Judit Serra-Pladevall, Luisa Pedro-Botet, Esteban Reynaga

**Affiliations:** 1 Department of Medicine, Universitat Autònoma de Barcelona, 08916 Badalona, Spain; mserras@fphag.org (M.S.-P.); jserrap@chv.cat (J.S.-P.); mlpbotet.germanstrias@gencat.cat (L.P.-B.); 2 Emergency Department, Hospital General de Granollers, 08402 Granollers, Spain; 3 Clinical and Environmental Infectious Diseases Study Group, Germans Trias i Pujol Research Institute, 08916 Badalona, Spain; nparraga@igtp.cat (N.P.-N.); earque@igtp.cat (E.A.); 4 Microbiology Department, Hospital Universitari de Vic, 08500 Vic, Spain; mnavarro@chv.cat (M.N.); avilamalab@chv.cat (A.V.); 5 Epidemiology Department, Hospital Universitari de Vic, 08500 Vic, Spain; epuigoriol@chv.cat; 6 Fundació Lluita Contra les Infeccions, Infectious Diseases Department, Hospital Universitari Germans Trias i Pujol, 08916 Badalona, Spain

**Keywords:** antimicrobial resistance, ESBL-producing *Escherichia coli*, prevalence, genomic, rectal colonization, livestock, Spain

## Abstract

Background and objectives: Extended-spectrum β-lactamase (ESBL)- and carbapenemase-producing Enterobacteriaceae represent an increasing One Health concern because food-producing animals may act as reservoirs of antimicrobial-resistant bacteria with potential transmission to humans. Therefore, this study aimed to assess the prevalence and genomic characteristics of ESBL- and carbapenemase-producing Enterobacteriaceae in livestock and farmers, and to evaluate genomic overlap between animal and human ESBL-producing *Escherichia coli* to explore potential shared origins and interspecies transmission. Methods: We conducted a cross-sectional study in Osona (Catalonia, Spain), sampling livestock (swine, cattle, poultry, and horses) and farmers. ESBL-, AmpC-, and carbapenemase-producing Enterobacteriaceae were identified and characterized using whole genome sequencing (WGS). Genomic analyses included sequence typing (ST), serotyping, virulence-associated genes, antimicrobial resistance determinants, and plasmid replicon profiling. Results: A total of 945 animals were analysed. ESBL-producing Enterobacteriaceae were predominantly detected in swine, with 63.5% (127/200) of animals testing positive, including four ESBL + AmpC-producing isolates and two carbapenem-resistant Enterobacteriaceae. No ESBL-, AmpC- or carbapenemase-producing isolates were identified in cattle (0/360) or poultry (0/171), and a low prevalence was observed in horses (7.0%, 15/214). A total of 64 farm workers were analysed. ESBL-producing *E. coli* were detected in 46.7% (7/15) of swine farmers and 8.3% (2/24) of cattle farmers, while no ESBL-producing isolates were found among poultry farmers (0/8) or equine caretakers (0/17). A total of 162 isolates were analyzed by WGS, showing high genetic diversity. Among the 127 *Escherichia coli* isolates, the most prevalent were ST10 (18/127, 14.2%), followed by ST453 (7/127, 5.5%), ST515 (7/127, 5.5%), and ST10562 (7/127, 5.5%). Virulence-associated gene profiles were heterogeneous, although genes related to stress tolerance and intestinal colonization predominated among ESBL-producing *E. coli* isolates from swine, including *terC* (54/127, 42.5%), *csgA* (41/127, 32.3%), *nlpI* (41/127, 32.3%), and *fimH* (39/127, 30.7%). ESBL production among *E. coli* isolates from swine was primarily mediated by *bla*CTX-M genes (89/127, 70.1%), with additional contributions from *bla*SHV (34/127, 26.8%) and *bla*TEM (40/127, 31.5%). Among swine isolates, IncX1 (100/127, 78.7%), IncFIB(AP001918) (82/127, 64.6%), IncI1-I(Alpha) (69/127, 54.3%), and IncFII (54/127, 42.5%) were the most frequently detected plasmid replicons. Two carbapenem-resistant isolates carrying *bla*OXA-48 were identified in swine, including *E. coli* ST58 and *K. oxytoca* ST145, both associated with IncL plasmids. Conclusions: Swine appear to constitute the primary reservoir of ESBL-producing *E. coli*. The genomic relatedness observed between animal and human isolates supports shared exposure to a common ecological pool of multidrug-resistant bacteria. The identification of *bla*OXA-48-producing Enterobacterales associated with IncL plasmids further highlights the public health relevance of livestock-associated antimicrobial resistance. However, the cross-sectional design precludes inference of transmission pathways or transmission directionality. Longitudinal studies are needed to elucidate the dynamics of interspecies transmission.

## 1. Introduction

Antimicrobial resistance (AMR) is a threat to global health and development, contributing to millions of deaths worldwide each year [1,2]. The misuse and overuse of antimicrobials in humans, animals, and plants are the main drivers in the development of drug-resistant pathogens [1]. In response, international organizations have promoted One Health strategies to address AMR, while resistant Gram-negative bacteria, including third-generation cephalosporin-resistant Enterobacterales (3GCRE) and carbapenem-resistant Enterobacterales (CRE), have been consistently identified among the highest public health priorities because of their global spread, limited therapeutic options and capacity to disseminate resistance determinants [3,4,5,6,7].

Members of the family Enterobacteriaceae, within the order Enterobacterales, particularly *Escherichia coli*, function as opportunistic pathogens that commonly inhabit the intestinal tract of humans and animals as commensals and are implicated in a wide range of community- and hospital-acquired infections. The presence of 3GCRE, usually due to the production of extended-spectrum β-lactamases (ESBLs) or AmpC β-lactamases (AmpCs), and CRE, due to the production of carbapenemase enzymes (CP), in the intestinal flora of animals, is undesired, due to the potential risk of transmission of resistant bacteria from animals and food to humans [8,9,10].

Globally, intensive farming is common, leading to cramped conditions, limited mobility, and the risk of contact with sick animals. In high-income countries, intensive pig production dominates and pigs are generally kept in indoor systems in which there is limited space, few opportunities to express natural behaviors, and many stressors that increase the risk of production diseases and infections, which leads to use and misuse of antibiotics [11]. Intensive pig production systems constitute a particularly relevant ecological niche for the emergence and maintenance of ESBL-producing *Escherichia coli*. High animal density, frequent antimicrobial exposure, and close contact between animals and farm workers create conditions that favour bacterial persistence, selection, and dissemination [8,11]. In this context, *E. coli*, as a commensal organism of the intestinal microbiota, plays a key role as a reservoir of antimicrobial resistance determinants [12].

Importantly, ESBL genes are frequently located on mobile genetic elements, especially conjugative plasmids, which facilitate horizontal gene transfer across bacterial populations and host species [12,13]. These plasmids often co-harbour additional antimicrobial resistance and virulence-associated genes, promoting both adaptation to the intestinal environment and survival under selective pressure [12,13]. Consequently, pig farms may act not only as reservoirs but also as amplification points for multidrug-resistant *E. coli* with the potential to spread through direct contact, environmental contamination, and the food chain [8,9,10].

Osona, an area of Barcelona province (Catalonia, northeast Spain), is a county with a high density of pig farms and high employment in this sector, representing one of the highest-density pig populations per km^2^ in the country [14,15], but also a moderate amount of cattle, poultry, and equine farms.

The aims of our study were: (a) to analyze the prevalence of ESBL- and carbapenemase-producing Enterobacteriaceae in the most common livestock species in the county (swine, cattle, poultry, and equine) and in the humans responsible for their care (farmers); (b) to genetically characterize any ESBL- and carbapenemase-producing Enterobacteriaceae strains detected; and (c) to compare ESBL-producing *E. coli* strains between animals and between animals and farmers to determine whether they shared a common origin and whether interspecies or zoo-anthropomorphic transmission might exist.

## 2. Material and Methods

We conducted a cross-sectional prevalence study in the county of Osona, Barcelona province, Catalonia, Northeast Spain (Figure S1), from January 2023 to December 2023.

### 2.1. Selection of Farms

Through the Livestock and Animal Health Section of Central Catalonia, we contacted swine, cattle, poultry, and equine veterinarians, who facilitated access to the corresponding farms. Animals were sampled as part of routine veterinary surveillance programs, and the vast majority were clinically healthy at the time of sampling. No systematic inclusion of animals with clinical disease, such as diarrhoeal symptoms, was performed. Therefore, the findings of this study primarily reflect colonization patterns in apparently healthy livestock rather than infection-related dynamics. We visited nine swine farms randomly distributed across the county of Osona, where samples were collected from 200 animals and 15 farmers; 10 cattle farms, where samples were obtained from 360 animals and 24 farmers; 11 equine facilities, where samples were collected from 214 horses and 17 caretakers; and five poultry farms, where samples were collected from 171 animals and eight farmers. In total, 35 farms were visited, and samples were obtained from 945 animals and 64 humans. The number of livestock and farmer samples collected from each farm is detailed in Table S1.

### 2.2. Collection of Animal Samples

A rectal swab was obtained from each animal using sterile cotton-tipped swabs and placed in Stuart swab PS+ Viscose (Deltalab, Rubí, Spain) [16]. All samples were stored at 4 °C and transported directly to the Microbiology Department of the Hospital Universitari de Vic (Vic, Spain) for analysis.

Sample collection was carried out by veterinary practitioners as part of the usual screening scheme conducted on farms, strictly adhering to Spanish ethical guidelines and animal welfare regulations (Real Decreto 53/2013). The collection of this material, considered routine veterinary practice, did not require the approval of the Ethics Committee for Animal Experimentation. Informed oral consent was obtained from the farmers at the time of sample collection.

### 2.3. Collection of Human Samples

All workers aged ≥18 years who were present at the time of the visit were invited to participate in the study, and all participants provided written informed consent. The study was approved by the Clinical Research Ethics Committee of the Consorci Hospitalari de Vic, the referral hospital for the Osona region, where sampling was conducted (approval no. 2021165). Basic demographic data were collected from all participants. Faecal samples were collected in sterile containers, stored at 4 °C, and transported directly to the Department of Microbiology of the Hospital Universitari de Vic (Vic, Spain) for analysis.

### 2.4. Microbiological Analysis of Samples

All samples were plated on selective media, namely MacConkey agar, CHROMID^®^ESBL (BioMerieux^®^, Marcy-l’Étoile, France) and CHROMID^®^CARBA SMART (BioMerieux^®^) [17]. The antimicrobial susceptibility of all enterobacterial isolates recovered on CHROMID^®^ESBL (BioMerieux^®^) and CHROMID^®^CARBA SMART (BioMerieux^®^) was studied by the microdilution method using the Vitek2 (BioMerieux^®^). The compounds studied were ampicillin, amoxicillin-clavulanate, piperacillin-tazobactam, cefuroxime, cefoxitin, ceftazidime, ceftriaxone, cefepime, ertapenem, meropenem, ciprofloxacin, gentamicin, amikacin, trimethoprim-sulfamethoxazole, nitrofurantoin, and fosfomycin. Interpretation of the results was performed according to the EUCAST clinical breakpoints when available [17].

Extended-spectrum β-lactamase and carbapenemase-producing Enterobacteriaceae strains were frozen at −20 °C and then sent to Germans Trias i Pujol Research Institute (IGTP) for whole genome sequencing.

### 2.5. DNA Extraction and Quantification

DNA extraction of all isolated bacterial strains was performed using the QIAamp DNA Blood Mini Kit (QIAGEN, Hilden, Germany) following the manufacturer’s instructions. Extractions were quantified by fluorometry (Quantus, Promega, Madison, WI, USA).

### 2.6. Whole Genome Sequencing

One hundred and sixty-two strains of Enterobacteriaceae were analyzed by Whole Genome Sequencing (WGS). DNA extractions were normalized at 0.2 ng/μL for library preparation with the Nextera XT DNA Library Preparation Kit (Illumina, San Diego, CA, USA). After the amplification step, the samples were purified with CleanNGS beads (CleanNA, Waddinxveen, The Netherlands). Quality control of libraries was performed using a 2200 TapeStation System (Agilent, Santa Clara, CA, USA). Libraries were individually quantified by fluorimetry (Quantus, Mascot, Australia), pooled and run on the MiSeq system (at 10 pM final concentration containing 10% PhiX) and a million reads per sample were obtained. Sequencing was performed at the Genomic Core Facility at the Centre de Regulació Genòmica in Barcelona.

Raw sequences were imported as paired-end sequences into the KBase platform [18]. Reads were de novo assembled using SPAdes assembler v3.13.0, with default parameters, to obtain contigs as FASTA files. These contigs were used to genotype isolates using Multi-Locus Sequence Typing (MLST) [19] and other tools available from the Center for Genomic Epidemiology (Technical University of Denmark), such as ResFinder version 4.7.2, FimTyper version 1.0, PlasmidFinder version 2.1, and VirulenceFinder version 2.0.

Relation among isolates was assessed using the goeBURST algorithm implemented in PHYLOViZ Online. Analysis was performed using MLST-derived allelic profiles. Network visualizations were generated using the default goeBURST settings.

### 2.7. Statistical Analysis

Statistical analysis was performed using IBM SPSS Statistics software version 30.0. Categorical variables were expressed as frequencies and percentages. The chi-square test (Fisher’s exact test) and Student’s *t*-test were used to compare the epidemiological characteristics between animal species, occupational groups, and positivity status. Statistical significance was set at *p* < 0.05.

## 3. Results

### 3.1. Prevalence of ESBL/AmpC- and Carbapenemase-Producing Enterobacteriaceae

A total of 64 farmers representing four livestock sectors were enrolled.

A total of 945 animals were sampled. The prevalence of ESBL/AmpC- and carbapenemase-producing Enterobacteriaceae differed significantly across livestock species (*p* < 0.001). Swine showed the highest prevalence (127/200 [63.5%]), compared with horses (15/214 [7.0%]), cattle (0/360), and poultry (0/171). The prevalence of ESBL-producing Enterobacteriaceae was significantly higher among swine farmers than among all other livestock workers combined (7/15 [46.7%] vs. 2/49 [4.1%], *p* < 0.001)

ESBL/AmpC- and carbapenemase-producing Enterobacteriaceae were predominantly detected in swine, with 127/200 (63.5%) animals and 7/15 (46.7%) swine farmers testing positive. Among the 127 positive swine samples, most isolates were ESBL-producing Enterobacteriaceae (121/127, 95.3%); four (3.1%) also harboured AmpC β-lactamases (ESBL + AmpC), and two (1.6%) were CRE, independent of ESBL production. Among swine farmers, 6 (85.7%) positive isolates were ESBL producers, and one (14.3%) ESBL + AmpC isolate.

All ESBL-producing isolates from swine and swine farmers were identified as *Escherichia coli*. Seven swine showed co-colonization with additional ESBL-producing Enterobacteriaceae, including *Klebsiella pneumoniae* (n = 3), *Klebsiella oxytoca* (n = 1), *Proteus hauseri* (n = 1), *Providencia rettgeri* (n = 1), and *Citrobacter freundii* (n = 1). One swine farmer was co-colonized with *Citrobacter freundii*.

In horses, 15/214 (7.0%) animals were positive for ESBL-producing Enterobacteriaceae, and all isolates were identified as *E. coli*, whereas no ESBL/AmpC- or carbapenemase-producing isolates were detected among equine caretakers (0/17). In cattle, no animals were positive (0/360), while ESBL-producing Enterobacteriaceae were detected in 2/24 cattle farmers (8.3%), and both isolates were identified as *E. coli*. In poultry, no animals (0/171) or farmers (0/8) were positive.

### 3.2. Comparative Phylogenomics

#### 3.2.1. Sequence Types

**Figure 1:**
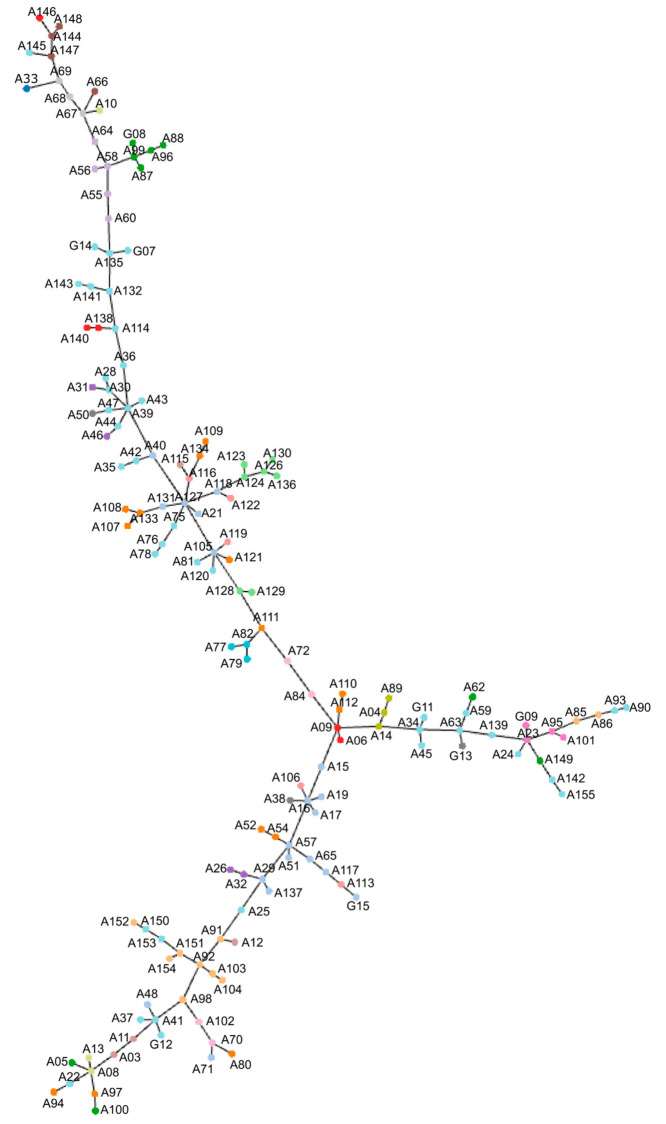
Relation among the isolates inferred from MLST data using the goeBURST algorithm. Nodes represent individual isolates. Colours indicate sequence type (ST) assignment, with isolates of the same ST shown in the same colour.

A wide diversity of sequence types (STs) was identified among ESBL-producing *E. coli* isolates, with no single ST predominating. Figure 1 presents the goeBURST network generated from MLST-derived allelic profiles, showing the relation among the isolates.

**Figure 2:**
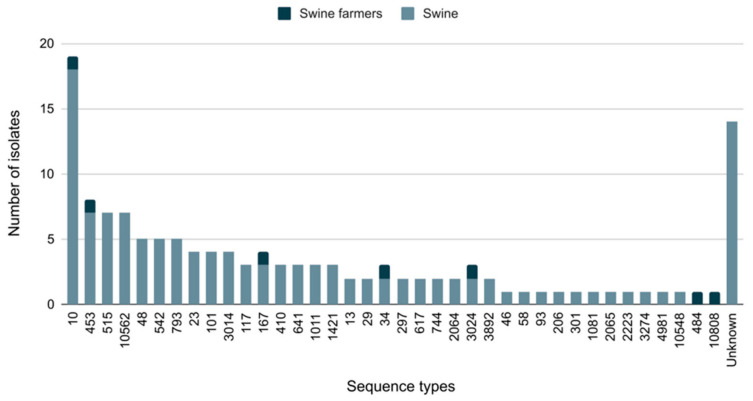
Distribution of the major sequence types (STs) of ESBL-producing *Escherichia coli* isolated from swine and swine farmers. Bars indicate the number of isolates according to host origin (swine vs. swine farmers).

Among swine isolates, the most frequent STs were ST10 (18/127, 14.2%), ST453 (7/127, 5.5%), ST515 (7/127, 5.5%), and ST10562 (7/127, 5.5%), although few isolates represented most STs. When restricting the comparison to swine and swine farmers, several STs were shared between the two groups, but none predominated in both populations. Fourteen isolates could not be assigned to a known ST (Figure 2).

*E. coli* isolates classified as ESBL + AmpC included ST515 (n = 1) and ST3892 (n = 2), whereas the single carbapenem-resistant *E. coli* isolate belonged to ST58.

Non-*E. coli* Enterobacteriaceae were identified in a limited number of cases (animals, n = 7; farmers, n = 1), comprising three *K. pneumoniae* isolates (all ST1564), one *K. oxytoca* isolate (ST145; CRE), two *C. freundii* isolates (both ST415; one recovered from swine and one from a swine farmer; ESBL + AmpC), one *P. rettgeri* isolate (ST unknown), and one *P. hauseri* isolate (ST unknown).

#### 3.2.2. Serotypes (O:H)

A high diversity of O and H antigens was observed among *E. coli* isolates, particularly in swine, with no single O:H serotype predominating. The most frequent O antigens in swine were O101 (15/127, 11.8%), O9a (14/127, 11.0%) and O23 (13/127, 10.2%), whereas H10 (27/127, 21.3%) and H45 (19/127, 15.0%) were the most common H antigens.

**Figure 3:**
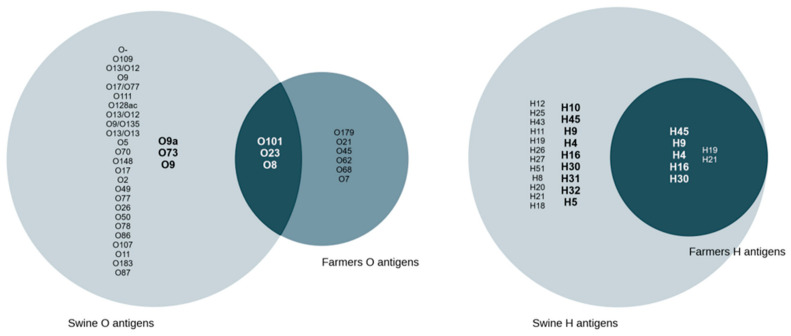
Distribution of shared and unique O (**left**) and H (**right**) antigens among ESBL-producing *Escherichia coli* isolates from swine and swine farmers. Only antigens detected in more than two isolates are shown. Antigens identified in more than ten isolates are highlighted in bold.

When restricting the comparison to swine and swine farmers, three O antigens (O101, O23 and O8) and eight H antigens were shared between the two groups, and all H antigens identified in human isolates were also detected in swine (Figure 3).

The single carbapenem-resistant *E. coli* isolate recovered from swine belonged to serotype O8:H10. Serotypes from minor hosts were heterogeneous, with no predominant pattern.

#### 3.2.3. Virulence-Associated Genes

Virulence-associated gene profiles were heterogeneous. Among ESBL-producing *E. coli* isolates from swine, genes related to stress tolerance and intestinal colonization predominated, including *terC* (54/127, 42.5%), *csgA* (41/127, 32.3%), *nlpI* (41/127, 32.3%), and *fimH* (39/127, 30.7%).

**Figure 4:**
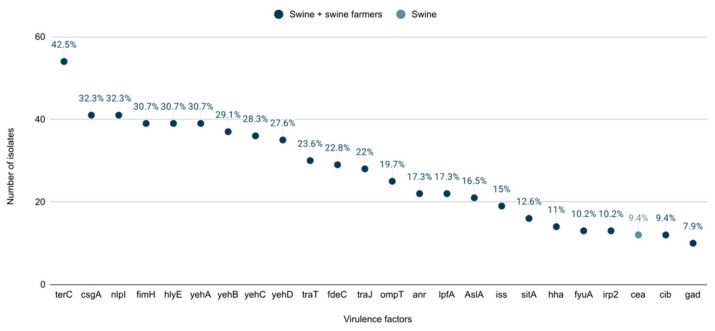
Virulence-associated genes detected in more than ten ESBL-producing *Escherichia coli* isolates from swine. Dark grey indicates genes also detected among isolates from swine farmers, whereas light grey indicates genes detected exclusively in swine isolates.

A substantial overlap was observed between swine and swine farmer isolates, as all but one of the virulence-associated genes detected in more than ten swine isolates were also identified among human isolates (Figure 4).

Isolates classified as ESBL + AmpC did not show distinct virulence patterns compared with ESBL-producing isolates.

The single carbapenem-resistant *E. coli* isolate recovered from swine harboured a broad repertoire of virulence-associated genes.

The complete distribution of virulence-associated genes across all isolates is provided in Table S2.

#### 3.2.4. Antimicrobial Resistance Genes

β-lactamase genes were widely distributed, reflecting the predominance of ESBL-producing isolates, with a smaller proportion of ESBL + AmpC co-producers and a limited number of carbapenem-resistant isolates. In addition to β-lactam resistance, genes conferring resistance to other antimicrobial classes were frequently detected, most commonly sulfonamides, trimethoprim, and fluoroquinolones, and only sporadically colistin and fosfomycin. Consequently, all isolates included in this study fulfilled criteria for multidrug resistance.

ESBL production among *E. coli* isolates from swine was primarily mediated by *blaCTX-M* genes (89/127, 70.1%), with additional contributions from *blaSHV* (34/127, 26.8%) and *blaTEM* (40/127, 31.5%). Resistance genes associated with all antimicrobial agents in swine *E. coli* isolates are presented in Table 1. 

AmpC β-lactamase genes were detected in a limited number of isolates and were restricted to swine. In *E. coli*, AmpC production was mediated by *blaDHA-1*, whereas AmpC-producing *C. freundii* isolates from swine and from one swine farmer carried *blaCMY-51*.

CRE was rare and confined to swine isolates, where it was mediated by *blaOXA-48* in both *E. coli* and *K. oxytoca*, in the absence of additional carbapenemase genes.

**Figure 5:**
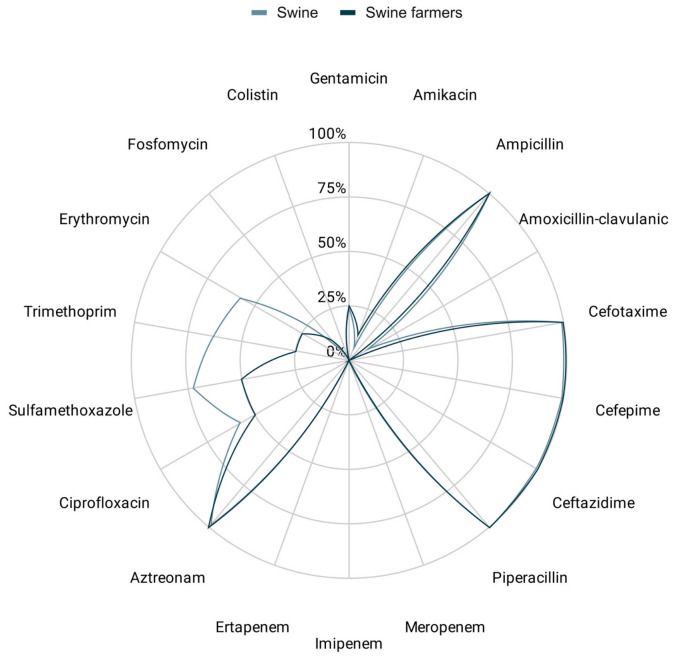
Genotypic resistance profiles of ESBL-producing *Escherichia coli* isolates recovered from swine and swine farmers. The figure illustrates the presence of antimicrobial resistance genes associated with resistance to the indicated antibiotic classes.

A broadly similar resistance gene repertoire was observed among *E. coli* isolates recovered from swine farmers. ESBL-associated genes and common co-resistance determinants largely overlapped those detected in swine isolates, despite the limited number of human samples. Consequently, genotypic resistance profiles were highly comparable between *E. coli* isolates recovered from swine and swine farmers (Figure 5).

Resistance genes detected in non-swine *E. coli* isolates and non-*E. coli* Enterobacterales are detailed in Table S3.

#### 3.2.5. Plasmid Replicon Types

Most ESBL-producing *E. coli* isolates harboured multiple plasmid replicons.

**Figure 6:**
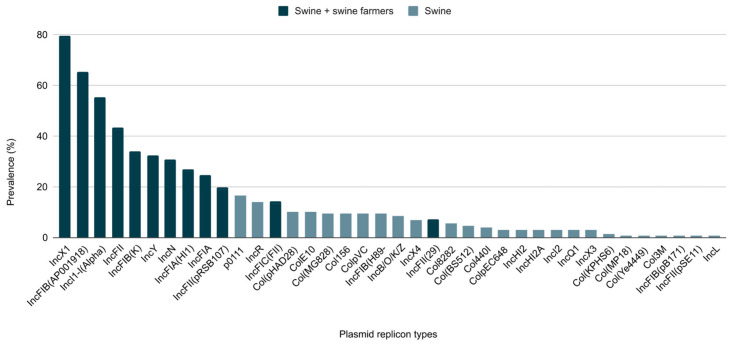
Distribution of plasmid replicon types among ESBL-producing *Escherichia coli* isolates from swine and swine farmers. Bars indicate the prevalence (%) of plasmid replicon types in swine isolates, with replicons also detected in swine farmer isolates highlighted in dark grey.

Among swine isolates, IncX1 (100/127, 78.7%), IncFIB(AP001918) (82/127, 64.6%), IncI1-I(Alpha) (69/127, 54.3%), and IncFII (54/127, 42.5%) were the most frequent replicons, frequently co-occurring within the same isolate. A comparable plasmid repertoire was observed among swine farmer isolates, with all replicon types detected in humans also present in swine (Figure 6).

ESBL genes were associated with diverse plasmid backbones. *blaCTX-M-1* was linked to IncX1, IncI1-I(Alpha), IncF and IncN plasmids, whereas *blaCTX-M-15* was mainly associated with IncF plasmids. *blaSHV-12* was also distributed across multiple plasmid families. In AmpC isolates, *blaDHA-1* co-occurred with IncFIB(AP001918), IncI1-I(Alpha), IncX1 and p0111 replicons, whereas *blaCMY-51* was associated with IncFII(Cf), IncHI1-related plasmids, IncN, IncP6 and IncX3. In CRE, *blaOXA-48* was associated with IncL plasmids in both *E. coli* and *K. oxytoca*.

The complete plasmid distribution is provided in Table S4.

## 4. Discussion

In this One Health study conducted in one of the most intensive livestock-producing regions of Spain, ESBL-producing Enterobacterales were detected predominantly in swine, whereas no ESBL-, AmpC-, or carbapenemase-producing isolates were identified in cattle or poultry and only a limited number were recovered from horses. These findings identify pig production systems as the principal reservoir of third-generation cephalosporin-resistant Enterobacterales in the study area and provide new insights into the genomic epidemiology of ESBL-producing *E. coli* at the animal–human interface.

Similar regional patterns have previously been described in the same geographical area for other antimicrobial-resistant bacteria, such as livestock-associated methicillin-resistant *Staphylococcus aureus* (LA-MRSA), highlighting the role of intensive pig production in the local epidemiology of antimicrobial resistance [14,15].

Furthermore, a recent study in the same region analysed both river and wastewater, detecting ESBL-producing *E. coli* in 5.6% and 18.5% of samples, respectively [20].

The high prevalence of ESBL-producing *E. coli* among swine farmers further supports the existence of close epidemiological links between occupational exposure and intestinal colonization by resistant bacteria. Nearly half of swine workers (46.7%) carried ESBL-producing *E. coli*, a prevalence substantially higher than that reported in other European countries. In a study from Denmark, the prevalence was 10% [21] and in the Netherlands ESBL-producing *E. coli* was detected in 17 of 146 farmers, family members and/or employees [22].

Although the cross-sectional nature of the study precludes establishing direct transmission events, the extensive genomic similarities observed between animal and human isolates suggest the possibility that farmers and livestock are exposed to a shared pool of multidrug-resistant bacteria within the production environment. Such exposure may occur through direct animal contact, contaminated farm environments, or indirect environmental pathways [23].

However, available evidence suggests that the emergence and maintenance of resistant *E. coli* in livestock may be more closely related to production systems than to animal species per se [8,24,25]. Intensive livestock production may favour the emergence and persistence of antimicrobial resistance through a combination of factors, including high animal density facilitating bacterial transmission, antimicrobial selection pressure, and ecological conditions that promote horizontal gene transfer among intestinal bacteria [12,25]. European surveillance data indicate that resistance to the highest-priority critically important antimicrobials among *E. coli* is widely distributed across multiple livestock species, although prevalence varies between countries and production systems [8]. At the national level, studies conducted in Spain have reported high levels of third-generation cephalosporin-resistant *E. coli* in pig production systems, which are predominantly intensive, with prevalences of ESBL- and/or AmpC-producing isolates reaching 77.1% in fattening pigs in the most recent EFSA/ECDC surveillance report [8], while similar resistance patterns have also been described in intensively managed cattle farms both in Spain and in other European countries [9,24]. Together, these observations suggest that the predominance of swine observed in the present study likely reflects the local structure of livestock production in Osona, where pig farming is largely intensive, rather than an intrinsic association between pigs and antimicrobial resistance.

Genomic analysis revealed a high diversity of sequence types and serotypes among ESBL-producing *E. coli*, with no single lineage predominating. Such genetic heterogeneity is consistent with previous studies showing that ESBL-producing *E. coli* circulating in human, animal, and environmental reservoirs often belong to diverse genetic backgrounds rather than to a limited number of epidemic clones [12,13,26]. Despite this diversity, a clear overlap of sequence types, serotypes, and virulence-associated genes was observed between isolates from swine and swine farmers. Notably, virulence gene repertoires were largely composed of factors involved in intestinal colonization, adhesion, and stress tolerance, which are commonly associated with commensal or opportunistic *E. coli* populations [12,13]. The extensive overlap in genomic features between animal and human isolates therefore supports the existence of a shared ecological pool of multidrug-resistant *E. coli* at the animal–human interface. Because of the cross-sectional design of the study, direct transmission between animals and humans cannot be demonstrated; however, the genomic similarity between isolates suggests repeated exposure to common reservoirs within the production environment.

All ESBL-producing isolates fulfilled criteria for multidrug resistance, reflecting the frequent co-occurrence of resistance genes beyond β-lactams. Similar co-resistance patterns have been widely reported among ESBL-producing *E. coli* in livestock and human populations across Europe [8,9,12,13] and other world regions [10,11]. The predominance of plasmid replicons belonging to the IncF, IncI, and IncX families further highlights the importance of mobile genetic elements in the dissemination of resistance genes. These plasmid backbones are well-known vectors for ESBL determinants and have been repeatedly identified in *E. coli* from livestock, food products, and humans [12,13]. The extensive plasmid diversity observed in swine isolates, together with the overlapping plasmid repertoires detected in swine farmers, suggests that horizontal gene transfer mediated by conjugative plasmids may play a central role in maintaining and disseminating multidrug resistance within pig production systems.

Although carbapenem-resistant isolates were rare, the detection of *blaOXA-48* in both *E. coli* and *K. oxytoca* recovered from swine deserves particular attention. OXA-48 is one of the most clinically significant carbapenemases worldwide and is frequently associated with successful epidemic plasmids, particularly those belonging to the IncL family [27]. In our study, *blaOXA-48* was identified in two phylogenetically distinct bacterial species and associated with IncL plasmids, suggesting the potential involvement of horizontal gene transfer mechanisms rather than clonal spread alone. While the low number of isolates precludes definitive epidemiological conclusions, the presence of OXA-48-producing Enterobacterales in livestock is concerning from a One Health perspective, as food-producing animals may contribute to the maintenance and environmental dissemination of clinically relevant resistance determinants. Continuous genomic surveillance is therefore warranted to monitor the occurrence and spread of carbapenemase-producing Enterobacterales at the human–animal interface.

An unexpected finding of our study was the absence of ESBL-, AmpC-, or carbapenemase-producing *E. coli* among cattle and poultry samples. Although ESBL-producing Enterobacterales have been reported in both animal sectors in several countries, prevalence estimates vary considerably according to geographical location, farming systems, biosecurity practices, sampling strategies, and antimicrobial usage patterns [28,29]. Therefore, these results should be interpreted within the specific epidemiological context of the study area and should not be extrapolated to broader cattle or poultry populations. Previous studies have shown substantial geographic and host-associated variability in the distribution of ESBL-producing *E. coli*, highlighting the importance of local ecological and management factors in shaping resistance patterns [12]. In addition, accumulating One Health evidence suggests that the epidemiology of ESBL-producing *E. coli* may differ markedly between regions and livestock production systems [30].

The cross-sectional design of the present study provides only a temporal snapshot of colonization and may not capture intermittent or low-frequency carriage events. Although selective culture methods were used, low-prevalence colonization cannot be completely ruled out.

This study has several limitations. First, its cross-sectional design provides a snapshot of colonization and does not allow assessment of temporal dynamics or determination of the direction of transmission between animals and humans. Second, the number of participating farmers was relatively small, particularly among swine workers, which may have limited the precision of prevalence estimates and reduced the ability to detect less frequent transmission events. Third, the study was conducted in a single high-density livestock region, which may affect the generalizability of the findings to other geographical settings with different farming practices.

In addition, the study focused primarily on colonization in apparently healthy animals and humans, and did not include a systematic evaluation of clinically ill animals, which may represent different epidemiological scenarios with potentially increased bacterial shedding and transmission. Future research should address these limitations through longitudinal study designs that enable the assessment of transmission dynamics over time and across different ecological compartments. Expanding the sample size and including more diverse human and animal populations would improve the robustness and external validity of the findings. Furthermore, the application of long-read sequencing technologies would allow more detailed characterization of plasmid structures, facilitating a better understanding of horizontal gene transfer and the dissemination of antimicrobial resistance within and between species. Integrating environmental sampling (e.g., water, soil, and farm surfaces) would also provide a more comprehensive view of the transmission pathways within a One Health framework.

In conclusion, pig production systems appear to constitute the main reservoir of ESBL-producing Enterobacterales in the study area and support the circulation of a shared ecological pool of multidrug-resistant *E. coli* at the animal–human interface. Despite the marked genetic diversity observed, the extensive overlap in resistance determinants, virulence-associated genes, and plasmid repertoires between swine and swine farmers suggests exposure to common reservoirs within the production environment. The detection of *blaOXA-48*-carrying Enterobacterales associated with IncL plasmids highlights the potential emergence of clinically relevant carbapenem resistance mechanisms in livestock. These findings reinforce the need for integrated One Health surveillance targeting animal reservoirs, occupational exposure, and mobile genetic elements involved in antimicrobial resistance dissemination.

## Figures and Tables

**Table 1:** Distribution of antimicrobial resistance genes detected by whole-genome sequencing in *Escherichia coli* isolates recovered from swine. Each panel represents a different antimicrobial agent. Bars indicate the prevalence (%) of resistance genes associated with the corresponding antibiotic among swine *Escherichia coli* isolates. Genes detected in at least one isolate are shown, and absence of known resistance genes is indicated as “No resistance”.

Resistance Gene	Gent.	Amik.	Ampi.	Amoxi-clavul.	Cefota.	Cefepi.	Cefta.	Pipera.	Mero.	Imipe.	Ertape.	Aztre.	Cipro.	Sulfa.	Trime.	Erythro.	Fosfo.	Colis.
No resistance	76.4	93.7	–	89.8	0.8	0.8	0.8	–	99.2	99.2	100	1.6	42.5	27.6	37	42.5	91.3	99.2
*aac(3)-IV*	15.7	–	–	–	–	–	–	–	–	–	–	–	–	–	–	–	–	–
*aac(3)-IId*	7.9	–	–	–	–	–	–	–	–	–	–	–	–	–	–	–	–	–
*aac(6′)-Ib-cr*	–	2.4	–	–	–	–	–	–	–	–	–	–	3.1	–	–	–	–	–
*aac(6′)-Ib3*	–	2.4	–	–	–	–	–	–	–	–	–	–	–	–	–	–	–	–
*aac(6′)-Ib-Hangzhou*	–	0.8	–	–	–	–	–	–	–	–	–	–	–	–	–	–	–	–
*aac(6′)-Ib-c*	–	0.8	–	–	–	–	–	–	–	–	–	–	–	–	–	–	–	–
*blaCTX-M-1*	–	–	24.4	–	35.4	35.4	35.4	24.4	–	–	–	35.4	–	–	–	–	–	–
*blaCTX-M-14*	–	–	3.9	–	6.3	8.7	8.7	3.9	–	–	–	8.7	–	–	–	–	–	–
*blaCTX-M-15*	–	–	11	1.6	11.8	11	11	11	–	–	–	11.9	–	–	–	–	–	–
*blaCTX-M-32*	–	–	6.3	–	11	11	11	6.3	–	–	–	10.2	–	–	–	–	–	–
*blaCTX-M-55*	–	–	0.8	–	2.4	2.4	2.4	0.8	–	–	–	2.4	–	–	–	–	–	–
*blaCTX-M-138*	–	–	0.8	–	1.6	1.6	1.6	0.8	–	–	–	1.6	–	–	–	–	–	–
*blaCTX-M-146*	–	–	1.6	–	1.6	1.6	1.6	1.6	–	–	–	1.6	–	–	–	–	–	–
*blaTEM-1A*	–	–	5.5	–	–	–	–	5.5	–	–	–	–	–	–	–	–	–	–
*blaTEM-1B*	–	–	22.8	0.8	–	–	–	22	–	–	–	–	–	–	–	–	–	–
*blaTEM-1C*	–	–	1.6	–	–	–	–	1.6	–	–	–	–	–	–	–	–	–	–
*blaTEM-40*	–	–	–	0.8	–	–	–	–	–	–	–	–	–	–	–	–	–	–
*blaTEM-206*	–	–	0.8	–	–	–	–	0.8	–	–	–	–	–	–	–	–	–	–
*blaSHV-12*	–	–	16.5	–	26.8	26.8	26.8	16.5	–	–	–	26.8	–	–	–	–	–	–
*blaDHA-1*	–	–	2.4	2.4	2.4	–	–	2.4	–	–	–	–	–	–	–	–	–	–
*blaOXA-1*	–	–	0.8	3.9	–	0.8	0.8	1.6	–	–	–	–	–	–	–	–	–	–
*blaOXA-48*	–	–	0.8	9.8	–	–	–	0.8	0.8	0.8	–	–	–	–	–	–	–	–
*qnrS1*	–	–	–	–	–	–	–	–	–	–	–	–	44.1	–	–	–	–	–
*qnrS13*	–	–	–	–	–	–	–	–	–	–	–	–	1.6	–	–	–	–	–
*qnrB4*	–	–	–	–	–	–	–	–	–	–	–	–	2.4	–	–	–	–	–
*qnrB19*	–	–	–	–	–	–	–	–	–	–	–	–	3.1	–	–	–	–	–
*qnrB55*	–	–	–	–	–	–	–	–	–	–	–	–	0.8	–	–	–	–	–
*qnrB81*	–	–	–	–	–	–	–	–	–	–	–	–	1.6	–	–	–	–	–
*qnrD1*	–	–	–	–	–	–	–	–	–	–	–	–	0.8	–	–	–	–	–
*sul1*	–	–	–	–	–	–	–	–	–	–	–	–	–	13.4	–	–	–	–
*sul2*	–	–	–	–	–	–	–	–	–	–	–	–	–	16.5	–	–	–	–
*sul3*	–	–	–	–	–	–	–	–	–	–	–	–	–	42.5	–	–	–	–
*dfrA1*	–	–	–	–	–	–	–	–	–	–	–	–	–	–	18.9	–	–	–
*dfrA5*	–	–	–	–	–	–	–	–	–	–	–	–	–	–	1.6	–	–	–
*dfrA12*	–	–	–	–	–	–	–	–	–	–	–	–	–	–	34.6	–	–	–
*dfrA14*	–	–	–	–	–	–	–	–	–	–	–	–	–	–	0.8	–	–	–
*dfrA17*	–	–	–	–	–	–	–	–	–	–	–	–	–	–	5.5	–	–	–
*dfrA36*	–	–	–	–	–	–	–	–	–	–	–	–	–	–	1.6	–	–	–
*mph(A)*	–	–	–	–	–	–	–	–	–	–	–	–	–	–	–	33.9	–	–
*mef(B)*	–	–	–	–	–	–	–	–	–	–	–	–	–	–	–	3.9	–	–
*mef(C)*	–	–	–	–	–	–	–	–	–	–	–	–	–	–	–	18.1	–	–
*erm(N)*	–	–	–	–	–	–	–	–	–	–	–	–	–	–	–	0.8	–	–
*mph(G)*	–	–	–	–	–	–	–	–	–	–	–	–	–	–	–	0.8	–	–
*fosA3*	–	–	–	–	–	–	–	–	–	–	–	–	–	–	–	–	8.7	–
*mcr-9.2*	–	–	–	–	–	–	–	–	–	–	–	–	–	–	–	–	–	0.8

Abbreviations: Gent., gentamicin; Amik., amikacin; Ampi., ampicillin; Amoxi-clavul., amoxicillin-clavulanic acid; Cefota., cefotaxime; Cefepi., cefepime; Cefta., ceftazidime; Pipera., piperacillin; Mero., meropenem; Imipe., imipenem; Ertape., ertapenem; Aztre., aztreonam; Cipro., ciprofloxacin; Sulfa., sulfamethoxazole; Trime., trimethoprim; Erythro., erythromycin; Fosfo., fosfomycin; Colis., colistin.

## Data Availability

Due to confidentiality restrictions, the data used in this study are not publicly available.

## Supplementary Materials

The following supporting information can be downloaded at: https://www.mdpi.com/article/10.3390/antibiotics15070676/s1, Figure S1. Geolocation; Table S1. Sampling strategy; Table S2. Virulence-associated genes; Table S3. Antimicrobial resistance genes; Table S4. Plasmid replicon types.
