# Supplementary material for: Prevalence and Genomic Characterization of ESBL-Producing Escherichia coli in Livestock and Farmers in Catalonia, Spain
Source: Antibiotics (Basel). 2026 Jul 9;15(7):676. doi: 10.3390/antibiotics15070676 (PMC13403780; doi:10.3390/antibiotics15070676)
Supplement: Supplementary file 1 [file antibiotics-15-00676-s001.zip › antibiotics-4413628-supplementary.pdf]

Supplementary material

Prevalence and genomic characterization of ESBL-producing *Escherichia coli* in livestock and farmers in Catalonia, Spain

Marina Serras-Pujol, Noemí Párraga-Niño, Marian Navarro, Anna Vilamala, Emma Puigoriol, Elisenda Arqué, Judit Serra-Pladevall, Maria Luisa Pedro-Botet, Esteban Reynaga

Table of content

Figure S1. Geolocation ... 2
Table S1. Sampling strategy ... 3
Table S2. Virulence-associated genes ... 4
Table S3. Antimicrobial resistance genes ... 7
Table S4. Plasmid replicon types ... 10

Figure S1. Geolocation

Geographic location of the study area. The county of Osona is highlighted within Catalonia, northeast Spain.

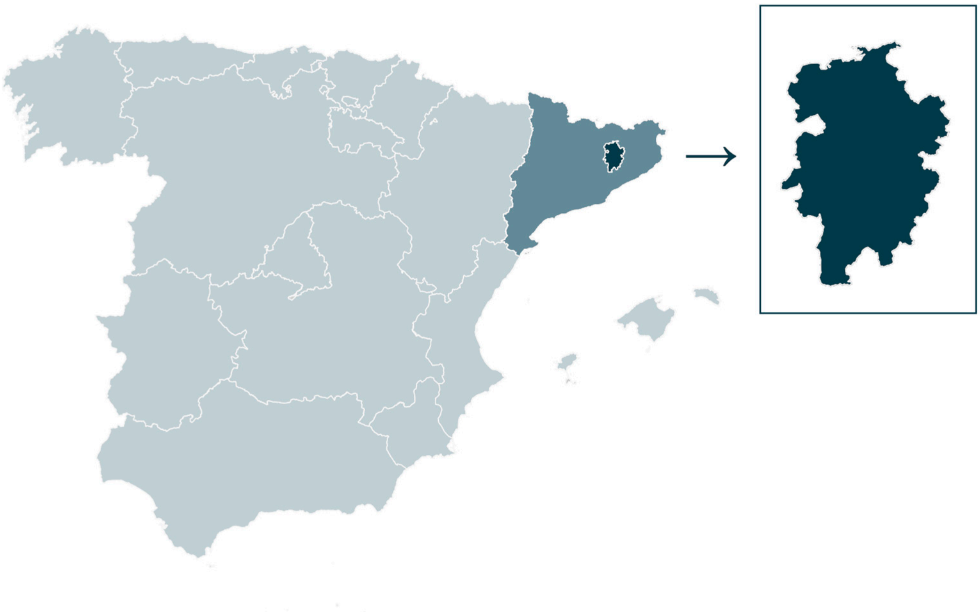

Table S1. Sampling strategy

Number of livestock and farmers sampled on each farm according to livestock species.

Swine		
Farm	No. livestock	No. farmers
1	20	7
2	20	1
3	20	1
4	20	2
5	20	1
6	40	1
7	20	1
8	20	1
9	20	0
	**200**	**15**

Cattle		
Farm	No. livestock	No. farmers
1	50	1
2	50	1
3	50	5
4	50	3
5	50	5
6	50	1
7	15	2
8	15	4
9	15	1
10	15	1
	**360**	**24**

Equine		
Farm	No. livestock	No. farmers
1	8	1
2	1	1
3	4	1
4	50	3
5	49	2
6	39	2
7	2	1
8	49	4
9	8	1
10	2	1
11	2	0
	**214**	**17**

Poultry		
Farm	No. livestock	No. farmers
1	21	2
2	60	2
3	30	1
4	30	2
5	30	1
	**171**	**8**

Table S2. Virulence-associated genes

Virulence-associated genes detected by whole-genome sequencing in ESBL- and CRE- Enterobacteriaceae isolates recovered from livestock and livestock farmers. Genes are grouped according to their main functional category based on standard virulence gene annotations. Values indicate the number of isolates positive for each gene within each group.

Abbreviations: CRE, carbapenem-resistant Enterobacterales; ESBL, extended-spectrum β-lactamase-producing; *E. coli*, *Escherichia coli*; *K. pneumoniae*, *Klebsiella pneumoniae*.

Category	Virulence-associated gene	Swine (*E. coli*) n=127	Swine farmers (*E. coli*) n=7	Horses (*E. coli*) n=15	Cattle farmer (*E. coli*) n=1	Swine (*K. pneumoniae*) n=3	Swine (CRE *E. coli*) n=1
Stress tolerance / environmental survival	terC	54	8	15	1	–	1
	gad	10	3	11	1	–	1
	hha	14	1	4	1	–	1
	anr	22	5	9	1	–	1
Adhesins / colonization factors	fimH	39	6	13	1	3	1
	csgA	41	7	13	1	–	1
	fdeC	29	6	11	1	–	1
	lpfA	22	3	11	1	–	1
	papC	6	3	1	1	–	–
	papA_F14	2	–	–	–	–	–
	papA_F43	–	–	–	1	–	–
	papA_F7-1	–	–	–	1	–	–
	yehA	39	7	15	1	–	1
	yehB	37	6	15	1	–	1
	yehC	36	6	15	1	–	1
	yehD	35	5	15	1	–	1
	mrkA	1	–	–	–	3	–
	tia	6	–	–	1	–	–
	hra	4	2	1	–	–	1
	iha	2	1	1	–	–	–
	tibC	1	–	–	–	–	–
Iron acquisition systems	fyuA	13	3	5	1	–	1
	irp2	13	2	5	1	–	1

	iroN	7	2	1	1	–	1
	iucC	9	1	4	1	–	1
	iutA	9	1	4	1	3	1
	sitA	16	3	5	1	–	1
	chuA	1	–	–	1	–	–
	ireA	1	–	–	1	–	–
Serum resistance / immune evasion	iss	19	5	6	1	–	1
	traT	30	5	9	1	–	1
	kpsE	6	3	1	1	–	–
	kpsMII_K5	4	–	–	1	–	–
	kpsMII_K23	1	1	–	–	–	–
	capU	7	1	3	–	–	–
Toxins and bacteriocins	hlyE	39	7	14	1	–	1
	hlyF	6	–	4	–	–	1
	hlyA	2	1	–	1	–	–
	cnf1	2	–	–	–	–	–
	astA	4	1	2	–	–	–
	sat	–	–	–	1	–	–
	cea	12	–	4	–	–	–
	cib	12	1	–	–	–	–
	cba	3	1	–	–	–	–
	cia	3	1	2	–	–	1
	cma	3	1	1	–	–	–
	colE5	1	–	1	–	–	–
	colE6	3	–	–	–	–	–
	colE7	–	–	–	1	–	–
	colE2-like	–	–	–	–	–	1
Other / miscellaneous	nlpI	41	7	15	1	–	1
	ompT	25	4	5	1	–	1
	traJ	28	5	6	1	–	1
	etsC	7	–	–	–	–	1
	tsh	5	–	1	–	–	–
	shiA	3	1	–	–	–	1
	shiB	5	1	3	1	–	–
	mchB	2	–	–	–	–	–
	mchC	2	–	–	–	–	–
	mchF	7	–	–	–	–	1

	yghJ	–	–	9	–	–	–
	fanA	–	–	1	–	–	–
	fanH	–	–	1	–	–	–
	estap-STa1	–	–	1	–	–	–
	sepA	–	–	1	–	–	–
	espP	1	–	1	–	–	–
	espY2	1	–	–	–	–	–
	etpD	1	–	–	–	–	–
	katP	1	–	–	–	–	–
	air	–	–	–	1	–	–
	eilA	–	–	–	1	–	–
	aamR	–	–	–	1	–	–

Table S3. Antimicrobial resistance genes

Distribution of antimicrobial resistance genes detected by whole-genome sequencing in isolates excluding swine *Escherichia coli*. The table summarises genotypic resistance determinants across isolates from other livestock species (first table) and farmers (second table). Resistance genes are reported by antimicrobial class, and absence of known resistance genes is indicated as “–”.

Abbreviations: AMR, antimicrobial resistance; CRE, carbapenem-resistant Enterobacterales; ESBL, extended-spectrum β-lactamase-producing; Gent., gentamicin; Amik., amikacin; Ampi., ampicillin; Amoxi-clavul., amoxicillin-clavulanic acid; Cefota., cefotaxime; Cefepi., cefepime; Cefta., ceftazidime; Pipera., piperacillin; Mero., meropenem; Imipe., imipenem; Ertape., ertapenem; Aztre., aztreonam; Cipro., ciprofloxacin; Sulfa., sulfamethoxazole; Trime., trimethoprim; Erythro., erythromycin; Fosfo., fosfomycin; Colis., colistin.

Micro.	Livest.	Resist. profile	Gent.	Amik.	Ampi.	Amoxi-clavul.	Cefota.	Cefepi.	Cefta.	Pipera.	Mero.	Imipe.	Ertape.	Aztre.	Cipro.	Sulfa.	Trime.	Erythro.	Fosfo.	Colis.
E. coli	Horse	ESBL	*aac(3)-IId*	–	*blaCTX-M-1*	–	*blaCTX-M-1*	*blaCTX-M-1*	*blaCTX-M-1*	*blaCTX-M-1*	–	–	–	*blaCTX-M-1*	*qnrS1*	*sul1*	*dfrA17*	–	–	–
E. coli	Horse	ESBL	–	–	*blaCTX-M-55*	–	*blaCTX-M-55*	*blaCTX-M-55*	*blaCTX-M-55*	*blaCTX-M-55*	–	–	–	*blaCTX-M-55*	–	–	–	–	–	–
E. coli	Horse	ESBL	*aac(3)-IId*	–	*blaCTX-M-1*	–	*blaCTX-M-1*	*blaCTX-M-1*	*blaCTX-M-1*	*blaCTX-M-1*	–	–	–	*blaCTX-M-1*	*qnrS1*	*sul1*	*dfrA12*	–	–	–
E. coli	Horse	ESBL	–	–	*blaCTX-M-15*	–	*blaCTX-M-15*	*blaCTX-M-15*	*blaCTX-M-15*	*blaCTX-M-15*	–	–	–	*blaCTX-M-15*	*qnrS1*	*sul2*	*dfrA14*	–	–	–
E. coli	Horse	ESBL	*ant(2")-Ia*	–	*blaTEM-1B*	–	*blaCTX-M-9*	*blaCTX-M-9*	*blaCTX-M-9*	*blaTEM-1B*	–	–	–	*blaCTX-M-9*	*qnrA1*	*sul2*	*dfrA1*	–	–	*mcr-9.2*
E. coli	Horse	ESBL	–	–	*blaOXA-10*	*blaTEM-30*	*blaCTX-M-65*	*blaCTX-M-65*	*blaCTX-M-65*	*blaOXA-10*	–	–	–	*blaOXA-10*	*qnrS1*	–	*dfrA14*	–	–	–

E. coli	Horse	ESBL	–	–	*blaCTX-M-15*	–	*blaCTX-M-15*	*blaCTX-M-15*	*blaCTX-M-15*	*blaCTX-M-15*	–	–	–	*blaCTX-M-15*	*qnrS1*	*sul2*	*dfrA1*	–	–	–
E. coli	Horse	ESBL	–	–	*blaCTX-M-15*	–	*blaCTX-M-15*	*blaCTX-M-15*	*blaCTX-M-15*	*blaCTX-M-15*	–	–	–	*blaCTX-M-15*	*qnrS1*	*sul2*	*dfrA14*	–	–	–
E. coli	Horse	ESBL	–	–	*blaCTX-M-14*	–	*blaCTX-M-14*	*blaCTX-M-14*	*blaCTX-M-14*	*blaCTX-M-14*	–	–	–	*blaCTX-M-14*	–	–	–	–	–	–
E. coli	Horse	ESBL	–	–	*blaSHV-12*	–	*blaSHV-12*	*blaSHV-12*	*blaSHV-12*	*blaSHV-12*	–	–	–	*blaSHV-12*	–	*sul1*	*dfrA17*	*mph(A)*	*fosA7*	–
E. coli	Horse	ESBL	–	–	*blaSHV-12*	–	*blaSHV-12*	*blaSHV-12*	*blaSHV-12*	*blaSHV-12*	–	–	–	*blaSHV-12*	–	–	–	–	–	–
E. coli	Horse	ESBL	–	–	*blaTEM-1B*	–	*blaSHV-12*	*blaSHV-12*	*blaSHV-12*	*blaTEM-1B*	–	–	–	*blaSHV-12*	–	*sul1*	*dfrA17*	*mph(A)*	*fosA7*	–
E. coli	Horse	ESBL	–	–	*blaTEM-1B*	–	*blaSHV-12*	*blaSHV-12*	*blaSHV-12*	*blaTEM-1B*	–	–	–	*blaSHV-12*	–	–	–	–	*fosA7*	–
E. coli	Horse	ESBL	–	–	*blaSHV-12*	–	*blaSHV-12*	*blaSHV-12*	*blaSHV-12*	*blaSHV-12*	–	–	–	*blaSHV-12*	–	–	–	–	–	–
E. coli	Horse	ESBL	*aac(3)-IId*	–	*blaCTX-M-55*	–	*blaCTX-M-55*	*blaCTX-M-55*	*blaCTX-M-55*	*blaCTX-M-55*	–	–	–	*blaCTX-M-55*	*qnrS1*	*sul3*	–	–	–	–
K. pneumoniae	Swine	ESBL	*aac(3)-IId*	*aac(6')-Ib-cr*	*blaSHV-11*	*blaSHV-26*	*blaSHV-98*	*blaSHV-98*	*blaSHV-98*	*blaSHV-11*	–	–	–	*blaSHV-98*	*aac(6')-Ib-cr*	*sul2*	*OqxB*	*mph(A)*	*fosA7*	–
K. pneumoniae	Swine	ESBL	*aac(3)-IId*	*aac(6')-Ib-cr*	*blaSHV-11*	*blaSHV-26*	*blaSHV-98*	*blaSHV-98*	*blaSHV-98*	*blaSHV-11*	–	–	–	*blaSHV-98*	*aac(6')-Ib-cr*	*sul2*	*OqxB*	*mph(A)*	*fosA7*	–
K. pneumoniae	Swine	ESBL	*aac(3)-IId*	*aac(6')-Ib-cr*	*blaSHV-11*	*blaSHV-26*	*blaSHV-98*	*blaSHV-98*	*blaSHV-98*	*blaSHV-11*	–	–	–	*blaSHV-98*	*aac(6')-Ib-cr*	*sul2*	*OqxB*	*mph(A)*	*fosA6*	–

K. oxytoca	Swine	CRE	*aac(3)-IIa*	–	*blaOXY-2-10*	*blaOXY-2-10*	*blaOXY-2-10*	–	*blaOXY-2-10*	*blaOXY-2-10*	*blaOXA-48*	*blaOXA-48*	–	–	*qnrS1*	*sul1*	*dfrA17*	*mph(A)*	–	–
C. freundii	Swine	ESBL + AmpC	–	–	*blaCMY-51*	*blaCMY-51*	*blaCMY-51*	*blaSHV-12*	*blaCMY-51*	*blaCMY-51*	–	–	–	*blaSHV-12*	*qnrS1*	–	–	–	–	–
P. rettgeri	Swine	ESBL	*aac(3)-IIa*	–	*blaCTX-M-32*	–	*blaCTX-M-32*	*blaCTX-M-32*	*blaCTX-M-32*	*blaCTX-M-32*	–	–	–	*blaCTX-M-32*	*qnrD1*	–	*dfrA1*	–	–	–
P. hauseri	Swine	ESBL	*aac(3)-IV*	–	*blaCTX-M-1*	–	*blaCTX-M-1*	*blaCTX-M-1*	*blaCTX-M-1*	*blaCTX-M-1*	–	–	–	*blaCTX-M-1*	*qnrD1*	*sul1*	*dfrA1*	*mph(B)*	–	–

Micro.	Livest. farmer	Resist. profile	Gent.	Amik.	Ampi.	Amoxi-clavul.	Cefota.	Cefepi.	Cefta.	Pipera.	Mero.	Imipe.	Ertape.	Aztre.	Cipro.	Sulfa.	Trime.	Erythro.	Fosfo.	Colis.
E. coli	Cattle	ESBL	–	–	*blaTEM-1B*	–	*blaCTX-M-15*	*blaCTX-M-15*	*blaCTX-M-15*	*blaTEM-1B*	–	–	–	*blaCTX-M-15*	*qnrS1*	*sul1*	*dfrA7*	–	–	–
E. coli	Swine	ESBL	–	–	*blaCTX-M-15*	–	*blaCTX-M-15*	*blaCTX-M-15*	*blaCTX-M-15*	*blaCTX-M-15*	–	–	–	*blaCTX-M-15*	–	*sul3*	–	*mef(C)*	*fosA3*	–
E. coli	Swine	ESBL	*aac(3)-IV*	–	*blaCTX-M-14*	–	*blaCTX-M-14*	*blaCTX-M-14*	*blaCTX-M-14*	*blaCTX-M-14*	–	–	–	*blaCTX-M-14*	–	*sul3*	–	–	–	–
E. coli	Swine	ESBL	–	–	*blaTEM-1B*	–	*blaSHV-12*	*blaSHV-12*	*blaSHV-12*	*blaTEM-1B*	–	–	–	*blaSHV-12*	*qnrS1*	–	–	–	–	–
E. coli	Swine	ESBL	–	–	*blaCTX-M-32*	–	*blaCTX-M-32*	*blaCTX-M-32*	*blaCTX-M-32*	*blaCTX-M-32*	–	–	–	*blaCTX-M-32*	–	–	–	–	–	–
E. coli	Swine	ESBL	–	–	*blaTEM-1B*	–	*blaCTX-M-14*	*blaCTX-M-14*	*blaCTX-M-14*	*blaTEM-1B*	–	–	–	*blaCTX-M-14*	–	–	–	–	–	–

E. coli	Swine	ESBL	–	–	*blaCTX-M-15*	–	*blaCTX-M-15*	*blaCTX-M-15*	*blaCTX-M-15*	*blaCTX-M-15*	–	–	–	*blaCTX-M-15*	*qnrS1*	–	–	–	–	–
E. coli	Swine	ESBL	*aac(3)-IId*	*aac(3)-IId*	*blaTEM-1B*	–	*blaCTX-M-32*	*blaCTX-M-32*	*blaCTX-M-32*	*blaTEM-1B*	–	–	–	*blaCTX-M-32*	*qnrS1*	*sul1*	*dfrA17*	*mph(A)*	–	–
C. freundii	Swine	ESBL + AmpC	*aac(3)-IV*	–	*blaCMY-51*	–	*blaCMY-51*	*blaCMY-51*	*blaCMY-51*	*blaCMY-51*	–	–	–	*blaSHV-12*	*qnrS1*	*sul1*	*dfrA12*	*msr(E)*	–	–

Table S4. Plasmid replicon types

Distribution of plasmid replicon types among ESBL-producing and carbapenem-resistant Enterobacteriaceae isolates recovered from animals and livestock farmers. Numbers represent the count of isolates harbouring each plasmid replicon type, with percentages calculated within each host–species group. Plasmid replicons were detected using whole-genome sequencing data. The table includes *Escherichia coli* isolates from swine, swine farmers, horses, and a cattle farmer, as well as non-*Escherichia coli* ESBL-producing and carbapenem-resistant Enterobacteriaceae recovered from swine and swine farmers. Individual isolates may carry more than one plasmid replicon.

<u>Abbreviations</u>: CRE, carbapenem-resistant Enterobacterales; ESBL, extended-spectrum β-lactamase-producing; *E. coli*, *Escherichia coli*; *K. pneumoniae*, *Klebsiella pneumoniae*; *K. oxytoca*, *Klebsiella oxytoca*; *C. freundii*, *Citrobacter freundii*; *P. rettgeri*, *Providencia rettgeri*; *P. hauseri*, *Proteus hauseri*.

Plasmid replicon type	Swine E. coli (n=127)	Swine farmers E. coli (n=7)	Horses E. coli (n=15)	Cattle farmer E. coli (n=1)	Swine K. pneumoniae (n=3)	Swine P. rettgeri (n=1)	Swine P. hauseri (n=1)	Swine ESBL + AmpC C. freundii (n=2)	Swine farmer ESBL + AmpC C. freundii (n=1)	Swine CRE E. coli (n=1)	Swine CRE K. oxytoca (n=1)
IncX1	100 (78.7%)	5 (71.4%)	2 (13,3%)	–	–	–	–	–	1 (100%)	–	–
IncFIB(AP001918)	82 (64.6%)	5 (71.4%)	10 (66,7%)	1 (100%)	–	–	–	–	–	1 (100%)	–
IncI1-I(Alpha)	69 (54.3%)	3 (42.9%)	5 (33,3%)	–	–	–	–	–	–	–	–

IncFII	54 (42,5%)	3 (42.9%)	6 (40%)	1 (100%)	–	–	–	–	–	1 (100%)	–
IncFIB(K)	42 (33,1%)	1 (14,3%)	3 (20%)	–	3 (100%)	–	–	–	1 (100%)	–	1 (100%)
IncY	40 (31,5%)	1 (14,3%)	1 (6,7%)	–	–	–	–	–	–	–	1 (100%)
IncN	38 (29,9%)	2 (28,6%)	–	–	–	–	–	–	1 (100%)	–	–
IncFIA(HI1)	33 (26%)	1 (14,3%)	1 (6,7%)	–	–	–	–	–	–	–	–
IncFIA	30 (23,6%)	2 (28,6%)	–	1 (100%)	–	–	–	–	–	–	–
IncFII(pRSB107)	24 (18,9%)	1 (14,3%)	–	–	–	–	–	–	–	–	–
p0111	21 (16,5%)	–	1 (6,7%)	–	–	–	–	–	–	–	–
IncR	18 (14,2%)	–	–	–	–	–	–	–	–	–	–
IncFIC(FII)	17 (13,4%)	1 (14,3%)	4 (26,7%)	–	–	–	–	–	–	–	–
Col(pHAD28)	13 (10,2%)	–	–	–	–	–	–	–	–	1 (100%)	–
ColE10	13 (10,2%)	–	–	–	–	–	–	–	–	–	–
Col(MG828)	12 (9,4%)	–	–	–	–	–	–	–	–	–	–
Col156	12 (9,4%)	–	–	1 (100%)	–	–	–	–	–	1 (100%)	–
ColpVC	12 (9,4%)	–	–	–	–	–	–	–	–	1 (100%)	–
IncFIB(H89-PhagePlasmid)	12 (9,4%)	–	–	–	–	–	–	–	–	–	–
IncB/O/K/Z	11 (8,7%)	–	–	–	–	–	–	–	–	–	–
IncX4	9 (7,1%)	–	–	–	–	–	–	–	–	–	–
IncFII(29)	8 (6,3%)	1 (14,3%)	–	–	–	–	–	–	–	–	–
Col8282	7 (5,5%)	–	–	–	–	–	–	–	–	–	–

Col(BS512)	6 (4,7%)	–	–	–	–	–	–	–	–	–	–
Col440I	5 (3,9%)	–	3 (20%)	1 (100%)	–	–	–	–	–	–	–
ColpEC648	4 (3,1%)	–	–	–	–	–	–	–	–	–	–
IncHI2	4 (3,1%)	–	2 (13,3%)	–	–	–	–	–	–	–	1 (100%)
IncHI2A	4 (3,1%)	–	2 (13,3%)	–	–	–	–	–	–	–	1 (100%)
IncI2	4 (3,1%)	–	–	–	–	–	–	–	–	–	–
IncQ1	4 (3,1%)	–	2 (13,3%)	1 (100%)	–	–	–	–	–	–	–
IncX3	4 (3,1%)	–	–	–	–	–	–	1 (100%)	–	–	–
Col(KPHS6)	2 (1,6%)	–	–	–	–	–	–	–	–	–	–
Col(MP18)	1 (0,8%)	–	–	–	–	–	–	–	–	–	–
Col(Ye4449)	1 (0,8%)	–	–	–	–	–	–	–	–	–	–
Col3M	1 (0,8%)	–	–	–	–	1 (100%)	1 (100%)	–	–	–	–
IncFIB(pB171)	1 (0,8%)	–	–	–	–	–	–	–	–	–	–
IncFII(pSE11)	1 (0,8%)	–	–	–	–	–	–	–	–	–	–
IncL	1 (0,8%)	–	–	–	–	–	–	–	–	1 (100%)	1 (100%)
IncFII(pHN7A8)	–	–	1 (6,7%)	–	–	–	–	–	–	–	–
IncHI1A	–	–	1 (6,7%)	–	–	–	–	–	–	–	–
IncHI1B(R27)	–	–	1 (6,7%)	–	–	–	–	–	–	–	–
pKPC-CAV1321	–	–	–	–	3 (100%)	–	–	–	–	–	–
IncFII(Cf)	–	–	–	–	–	–	–	1 (100%)	–	–	–
IncHI1A(NMD-CIT)	–	–	–	–	–	–	–	–	1 (100%)	–	–

IncHI1B(pNDM-CIT)	–	–	–	–	–	–	–	–	1 (100%)	–	–
IncP6	–	–	–	–	–	–	–	–	1 (100%)	–	–
